# Appraisal of universal health insurance and maternal health services utilization: pre- and post-context of the Jaminan Kesehatan Nasional implementation in Indonesia

**DOI:** 10.3389/fpubh.2024.1301421

**Published:** 2024-03-14

**Authors:** Tati Rahmawati, Hui-Min Hsieh

**Affiliations:** ^1^Department of Public Health, Kaohsiung Medical University, Kaohsiung, Taiwan; ^2^Department of Medical Research, Kaohsiung Medical University Hospital, Kaohsiung, Taiwan; ^3^Department of Community Medicine, Kaohsiung Medical University Hospital, Kaohsiung, Taiwan; ^4^Center for Big Data Research, Kaohsiung Medical University, Kaohsiung, Taiwan; ^5^Research Center for Environmental Medicine, Kaohsiung Medical University, Kaohsiung, Taiwan

**Keywords:** National Social Security System, Jaminan Kesehatan Nasional, universal health coverage, maternal care, Demographic and Health Surveys (DHS)

## Abstract

**Introduction:**

The Indonesian government introduced universal health insurance through the National Social Security System (JKN) in 2014 to enhance overall healthcare. This study compares maternal health care (MHC) service utilization before and after JKN implementation in Indonesia.

**Method:**

Using 2012 and 2017 data from Indonesia Demographic and Health Surveys (DHS), we conducted a two-period cross-sectional design study following the Anderson model. We assessed how the JKN policy and population characteristics influenced healthcare utilization for women aged 15–49 who had given birth in the last 5 years. Multivariable logistic regression models were used to assess the impact of the JKN policy and related factors.

**Result:**

In two waves of Indonesia DHS with 14,782 and 15,021 subjects, this study observed a significant increase in maternal healthcare service utilization post-JKN implementation. Women were more likely to have at least four antenatal care visits (adjusted odds ratio, AOR = 1.17), receive skilled antenatal care (AOR = 1.49), obtain skilled birth assistance (AOR = 1.96), and access facility-based delivery (AOR = 2.45) compared with pre-JKN implementation.

**Conclusion:**

This study revealed a significant positive impact of JKN on enhancing MHS utilization. The introduction of universal health insurance coverage likely reduced financial barriers for specific demographics, resulting in increased service utilization. Our study may offer valuable insights for Asian countries with similar demographics and health insurance implementations.

## Introduction

More than three-quarters of the women in WHO member states receive maternal services yet the extent of intraregional inequality remains overwhelming (1). In 2020, the daily count of women succumbing to pregnancy and childbirth complications exceeded 800, with 95% of these fatalities occurring in low and lower-middle-income countries (2). Indonesia, among Southeast Asia nations, exhibits a significantly higher maternal mortality ratio, with 189 maternal deaths per 100,000 live births (3). Despite the recommendations for prenatal care, a study discovered that 91.2% of women received inadequate antenatal care, resulting in a 1.3 fold increase in the likelihood of labor complications (4). The rate of institutional childbirth rose from 22% in 1986 to 73% in 2012 (5). Nevertheless, the absence of a consistent reduction in maternal deaths stems from substandard service quality in both within primary care setting and hospitals (6, 7).

Persistent challenges in low and lower middle-income countries are linked to Sustainable Development Goals (SDGs) 3, which sets a target to decrease the global maternal mortality ratio to less than 70 per 100,000 live births by year 2030. Analogously, SDGs 5, which focuses on gender equality and women’s rights, shares a comparable focus (8). In addition, the World Health Organization is committed to ensuring comprehensive maternal health services through the Ending Preventable Maternal Mortality Strategy (8). This program considers various factors, including health systems, universal health coverage (UHC), and socioeconomic determinants. It also emphasizes policy measures to support family planning, healthy pregnancies, and safe childbirth (9).

Numerous initiatives were introduced to bolster maternal health services in Indonesia, including the global Safe Motherhood Initiative was formulated during a conference in Nairobi in 1987 (10), with Indonesia holding its first national seminar on safe motherhood in 1988 (11). Subsequently, the village midwife (*bidan di desa*) program was established. Providing trained midwives in village birth facilities (*polindes*). The Action to Cherish Mother (*Gerakan Sayang Ibu*) was launched in 1996 as a government-led effort to reduce maternal mortality. Despite this intervention, traditional birth attendance (*dukun*) remains prevalent at the community level. Traditional birth attendants (dukun) typically lack formal training in modern medicine, which may result in lower service quality (12).

In 2014, the Indonesian government implemented a UHC initiative known as National Social Security System (JKN), with the core objective of offering comprehensive coverage for all citizens, including access to maternity care (13). Indonesia achieved universal maternal health coverage through a range of channels, including Askes (public sector social insurance coverage), Jamsostek (private sector social insurance coverage), and Jampersal (childbirth assurance coverage). These programs were eventually consolidated into a single-payer system under JKN, providing a comprehensive maternal health benefit package (14). Furthermore, the government introduced Jamkesmas (non-contributory social insurance coverage for the impoverished) and Jamkesda (local government-funded social insurance), specifically targeting individuals who were not covered by existing insurance schemes (12). It aims to ensure that all participants are rounded up by fair and equitable services. Every citizen is required to pay a premium of 5% of monthly earnings for both public and private sector workers. However, economically disadvantaged individuals, underprivilege, or have a disability receive ongoing healthcare premium subsidies from either central or local governments, as stipulated in Legislation Number 101 of 2013. In the early semester of 2014, JKN participants reached a number of 124.55 million people and increased to 241.79 million in 2022 (15).

The earlier examination indicated a substantial enhancement in maternal health service utilization due to the implementation of JKN (16, 17). A recent study by Aryastami and Mubasyiroh (16) utilized data from the 2018 Basic Health Research of Riskesdas and suggested that women with health insurance may be more likely to utilize maternal health services optimally. Another paper by Anindya et al. (17) assessed the impact of Indonesia’s National Health Scheme on access to maternal health services. However, most of these studies relied on single-year cross-sectional data to investigate the influence of health insurance coverage on maternal health service utilization, rather than emphasizing the assessment of pre- and post-policy effects on care. Furthermore, there was a lack of information regarding the existence of variations in the utilization and readiness of maternal health services across different regions within Indonesia (14, 18).

This study utilized two waives of data from the Indonesia Demographic Health Surveys (DHS) conducted in 2012 and 2017, covering the period before and after the implementation of the universal health insurance system through the National Social Security System in Indonesia in 2014. The objective was to assess the influence of JKN on maternal health service (MHS) utilization, specifically to compare MHS utilization between the periods prior to (Phase 1 in 2012) and following (Phase 2 in 2017) the implementation of JKN. To delve further into this investigation on engaging policymakers and relevant stakeholders who can serve as conduits between various entities, stratification analyses were conducted to precisely determine the extent of MHS utilization before and after the introduction of JKN among different Indonesian women subgroups within the reproductive age group.

## Materials and methods

### Conceptual framework

**Figure 1:**
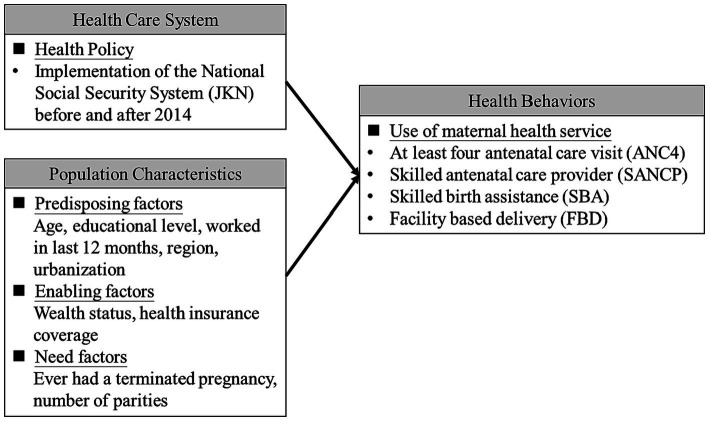
Conceptual framework of Anderson behavioral model in this study.

The Anderson behavioral model was adopted as the conceptual framework in this study for investigating factors associated with maternal health services utilizations. Many literatures have used this framework developed by Ronald M. Andersen in 1968 and advanced version for investigating individual and contextual determinants of health service use (19–22). As Figure 1 shows the conceptual framework for the association between health care system (before and after the implementation of JKN in 2014), population characteristics (predisposing factors, enabling factors, and needs) and utilization of maternal health services.

### Study setting, design and data source

Indonesia is an archipelagic nation situated in Southeast Asia, known as the world’s largest island nation, comprising over 17,000 islands. The five primary islands are Java, Sumatra, Borneo (Kalimantan), Sulawesi, and Papua, in addition to numerous smaller islands. Among women aged 15–49 in Indonesia, traditional patriarchal culture in Indonesia still exerts its influence on the choices and behaviors of a considerable number of Indonesian women in particular respects of their lives (23). Furthermore, there exists a notable variation in the educational attainment of this demographic (24). Women of maternal age are at a higher risk of experiencing unmet healthcare needs and may be less likely to receive adequate health services in Indonesia.

This study employed a two-period cross-sectional study design, utilizing two waves of data from the Indonesia Demographic Health Surveys (DHS) conducted in 2012 and 2017. These surveys spanned both pre- and post-implementation periods, allowing for an assessment of the JKN implementation. DHS surveys are conducted in collaboration between national government agencies and international organizations, providing comprehensive data on various demographic and health indicators in developing countries, making it a valuable resource for research and evaluate healthcare demand and provision for the improvement of women and children’s health. The survey datasets, accessible through an online application request for research and study purposes, have been approved by the International Coaching Federation (ICF) Institutional Review Board. Ethical clearance for the 2012 IDHS-VI and 2017 IDHS-VII was obtained from the National Institute of Health Research and Development, Indonesia Ministry of Health. Each request for data access is considered for a specific research project (25).^1^

### Study population and sampling technique

The two waves of Indonesia DHS included a representative population of 43,852 and 47,963 households nationwide, including all administrative regions of the country, and both urban and rural areas, respectively in all 33 provinces in 2012 and 34 provinces in 2017. A two-stage stratification cluster design prevailed to determine a study participant, the first stage was by selecting enumeration areas (EA) from census files, and the second stage was by selecting households’ samples in each EA selected to be interviewed.

This study only included women aged 15–49 who had given birth within the 5 years preceding two waves of the women’s questionnaire in the Indonesia DHS survey. To streamline the analysis, a dataset was generated by consolidating all pertinent information regarding their most recent childbirth. The final number of eligible subjects for analysis was 14,782 women in 2012 (phase 1) and 15,021 in 2017 (phase 2).

### Variable measurements

The outcomes of interest were MHS utilizations women aged 15 to 49 who had given birth within the 5 years in two waves of Indonesia DHS survey between the periods before (Phase 1 in 2012) and after (Phase 2 in 2017) JKN implementation. Specifically, the study included four binary variables for measuring MHS utilizations, including whether a woman received at least Four Antenatal Care (ANC4, yes/no), visited a Skilled Antenatal Care Provider (SANCP, yes/no), gave birth with Skilled Birth Assistance (SBA, yes/no), and utilized a Facility-Based Delivery (FBD, yes/no).

The primary explanatory variable of this study was the implementation of the JKN policy. The JKN implementation is categorized into pre-implementation (Phase1 in year 2012) and post-implementation (Phase 2 in year 2017). Furthermore, following the Anderson behavioral model, this study included predisposing indicators (age [<25/25–29/30–34/35–39/≥ 40], educational level [no education and primary education/ secondary education/ higher education], work status [yes/no], and regions [Java/Sumatra/ Bali and Nusa Tenggara/ Kalimantan/ Sulawesi/ Maluku and Papua], and urbanization [urban/rural]), enabling indicators (household wealth status [poor/ middle/ rich], and health insurance coverage [no covered/ covered]), and need indicators (ever had complications during pregnancy [yes/no], and number of parity [<2/> = 2]).

### Statistical analysis

This study employed weighted statistical calculations to account for population size and ensure data representativeness. Given the study’s focus on female respondents, we exclusively applied the weights designated for women. In the initial phase, a new variable was created by dividing a specific women-related variable (V005) from the DHS by 1,000,000, and this variable was subsequently used in tabulations. Percentage and frequency data were used to describe population characteristics and maternal health service (MHS) utilization, comparing the periods before (Phase 1 in 2012) and after (Phase 2 in 2017) JKN implementation. We used the Chi-square test to compare differences in characteristics and outcomes attributed to the JKN policy’s effects. To examine the association between the JKN policy effect and four MHS outcomes, while controlling for the population characteristics outlined in Table 1, we conducted multivariate logistic regressions. Adjusted odds ratios (AORs) and 95% confidence intervals (CIs) were reported. Furthermore, this study conducted stratified analyses to assess the JKN policy effect among various population subgroups, summarizing the results using forest plots. The study was conducted from September 2022 to July 2023. Data analysis for this research was carried out using IBM SPSS Statistics, Version 20.0, and statistical significance was considered for *p*-values <0.05.

**Table 1:** Demographic characteristics of respondents in Phase 1 (2012) and Phase 2 (2017).

Parameter	Phase 1 (2012)	Phase 2 (2017)	*p*-value^a^
*N*	14,782	15,021	
Predisposing [*n* (%)]			
Age (years)			
< 25	3,355 (22.70%)	2,856 (19.01%)	<0.001
25–29	4,063 (27.49%)	3,791 (25.24%)	
30–34	3,562 (24.10%)	3,833 (25.52%)	
35–39	2,495 (16.88%)	3,010 (20.04%)	
≥ 40	1,307 (8.84%)	1,531 (10.19%)	
Educational level			
No education and primary education	5,031 (34.03%)	4,064 (27.05%)	<0.001
Secondary education	7,987 (54.03%)	8,754 (58.28%)	
Higher education	1,764 (11.94%)	2,203 (14.67%)	
Worked in last 12 months			
No	6,856 (46.38%)	7,261 (48.34%)	0.001
Yes	7,926 (53.62%)	7,760 (51.66%)	
Region			
Java	8,145 (55.10%)	8,257 (54.97%)	0.652
Sumatera	3,317 (22.44%)	3,374 (22.46%)	
Bali and Nusa Tenggara	896 (6.06%)	946 (6.30%)	
Kalimantan	925 (6.26%)	952 (6.34%)	
Sulawesi	1,078 (7.29%)	1,036 (6.90%)	
Maluku and Papua	421 (2.85%)	456 (3.04%)	
Urbanization			
Urban	7,350 (49.72%)	7,284 (48.49%)	0.034
Rural	7,432 (50.28%)	7,737 (51.51%)	
Enabling [n (%)]			
Wealth status			
Poor	5,916 (40.02%)	6,008 (39.99%)	0.220
Middle	2,939 (19.88%)	3,099 (20.63%)	
Rich	5,927 (40.10%)	5,915 (39.38%)	
Health insurance coverage			
Noncovered	9,418 (63.71%)	6,213 (41.36%)	<0.001
Covered	5,364 (36.29%)	8,808 (58.64%)	
Need [n (%)]			
Ever had a terminated pregnancy			
No	12,779 (86.45%)	12,868 (85.67%)	0.051
Yes	2,003 (13.55%)	2,153 (14.33%)	
Number of parities			
> 2	5,226 (35.35%)	5,472 (36.43%)	0.053
≤ 2	9,556 (64.65%)	9,549 (63.57%)	

^a^Chi-square test to compare differences in characteristics and outcomes attributed to the JKN policy’s effects and *p*-value was presented.

## Results

Table 1 shows general characteristics of women who gave births in the last 5 years. A total of 14,782 women participated in phase 1, while 15,021 women were involved in phase 2. In phase 1, women aged 25–29 years old were the most prominent (*n* = 4,063, 27.49%), whereas in phase 2, those aged 30–34 years old were the largest group (*n* = 3,833, 25.52%). The majority of women in both phases had completed secondary education (*n* = 7,987, 54.03% in phase 1; *n* = 8,754, 58.28% in phase 2), were employed in the last 12 months (*n* = 7,926, 53.62% in phase 1; *n* = 7,760, 51.66% in phase 2), resided in rural areas (*n* = 7,432, 50.28% in phase 1; *n* = 7,737, 51.51% in phase 2). Furthermore, there was an increase in health insurance coverage from phase 1 (*n* = 5,364, 36.29%) to phase 2 to (*n* = 8,808, 58.64%).

Table 2 presents the distribution of women’s utilization of MHS before and after JKN implementation. There was an increase in the number of women receiving antenatal care ≥4 visits from 12,974 women (87.79%) in phase 1 with 110 missing values to 13,603 women (90.59%) in phase 2 with 65 missing values. Similarly, the proportion of respondents visiting skilled antenatal care providers increased from 14,147 women (95.70%) in phase 1 with 65 missing values to 14,647 women (97.50%) in phase 2 with 41 missing values. Additionally, the number of women giving birth with skilled birth assistance rose from 12,466 women (84.30%) in phase 1 with 60 missing values to 13,787 women (91.80%) in phase 2 with 36 missing values. Furthermore, the utilization of facility-based delivery increased from 9,541 women (64.79%) in phase 1 with 54 missing values to 12,076 women (80.59%) in phase 2 with 37 missing values. The study findings demonstrated a significant association between all maternal health services utilization and the two phases of cohorts (*p* < 0.001). Due to the small number of missing values, subjects with missing value were not considered and excluded for further analysis.

**Table 2:** Distribution of maternal health care services utilization.

Parameter	Phase 1 (2012)	Phase 2 (2017)	*p*-value^a^
Antenatal care			
<4 visits	1,698 (11.49%)	1,353 (9.01%)	<0.001
≥4 visits	12,974 (87.79%)	13,603 (90.59%)	
Missing	110 (0.72%)	65 (0.40%)	
Skilled antenatal care provider			
No	570 (3.86%)	333 (2.22%)	<0.001
Yes	14,147 (95.70%)	14,647 (97.50%)	
Missing	65 (0.44%)	41 (0.28%)	
Skilled birth assistance			
No	2,256 (15.30%)	1,198 (8.00%)	<0.001
Yes	12,466 (84.30%)	13,787 (91.80%)	
Missing	60 (0.40%)	36 (0.20%)	
Facility based delivery			
Home	5,186 (35.21%)	2,908 (19.41%)	<0.001
Health facilities	9,541 (64.79%)	12,076 (80.59%)	
Missing	54 (0.37%)	37 (0.25%)	

^a^Chi-square test to compare differences in characteristics and outcomes attributed to the JKN policy’s effects and *p*-value was presented.

Table 3 shows the multivariable logistic regression results for examining policy factors, predisposing, needs, and enabling factors associated with MHS utilization. With respect to the policy effect of the implementation of the JKN implementation (compared with Phase 1), Indonesian women were found to be more likely to have at least four antenatal care visits (aOR = 1.17, 95%CI = 1.07 ~ 1.27), received care from skilled antenatal care provider (aOR = 1.49, 95%CI = 1.29 ~ 1.73), receive skilled birth assistance (aOR = 1.96, 95%CI = 1.81 ~ 2.13), and accessed facility based delivery (aOR = 2.45, 95%CI = 2.30 ~ 2.60).

**Table 3:** Results from multivariable logistic regressions to examine policy Factors, predisposing, needs, and enabling Factors associated with four types of MHS utilization.

Variables	At least four antenatal care visits		Skilled antenatal care provider		Skilled birth assistance		Facility based delivery	
	AOR(95%CI)	*p*-value	AOR (95%CI)	*p*-value	AOR(95%CI)	*p*-value	AOR (95%CI)	*p*-value
Policy factor								
Pre-JKN Implementation	1.00		1.00		1.00		1.00	
Post-JKN Implementation	1.17 (1.07,1.27)	<0.001	1.49 (1.29,1.73)	<0.001	1.96 (1.81,2.13)	<0.001	2.45 (2.30,2.60)	<0.001
Predisposing								
Age (years)								
< 25	1.00		1.00		1.00		1.00	
25–29	1.18 (1.05,1.32)	0.005	1.15 (0.93,1.41)	0.188	1.11 (0.99,1.24)	0.068	1.00 (0.92,1.09)	0.957
30–34	1.22 (1.08,1.38)	0.001	1.07 (0.87,1.33)	0.527	1.15 (1.03,1.30)	0.015	1.04 (0.95,1.14)	0.351
35–39	1.01 (0.90,1.15)	0.817	0.85 (0.69,1.06)	0.157	1.39 (1.22,1.58)	<0.001	1.21 (1.10,1.33)	<0.001
≥ 40	0.82 (0.71,0.95)	0.008	0.70 (0.55,0.90)	0.005	1.18 (1.02,1.37)	0.025	1.05 (0.93,1.18)	0.431
Educational level								
No education and primary education			1.00		1.00		1.00	
Secondary education	2.10 (1.92,2.29)	<0.001	2.97 (2.53,3.48)	<0.001	3.13 (2.88,3.41)	<0.001	2.35 (2.20,2.51)	<0.001
Higher education	3.03 (2.51,3.67)	<0.001	5.41 (3.36,8.70)	<0.001	7.52 (5.83,9.70)	<0.001	3.78 (3.33,4.29)	<0.001
Worked in last 12 months								
No	1.00		1.00		1.00		1.00	
Yes	0.93 (0.85,1.00)	0.061	0.92 (0.80,1.06)	0.249	0.82 (0.75,0.88)	<0.001	0.88 (0.83,0.93)	<0.001
Region								
Java	1.00		1.00		1.00		1.00	
Sumatera	0.42 (0.38,0.46)	<0.001	0.44 (0.36,0.53)	<0.001	1.38 (1.23,1.53)	<0.001	0.45 (0.42,0.49)	<0.001
Bali and Nusa Tenggara	1.13 (0.93,1.36)	0.222	0.82 (0.60,1.13)	0.232	0.89 (0.77,1.04)	0.132	1.21 (1.06,1.37)	0.005
Kalimantan	0.61 (0.52,0.71)	<0.001	0.39 (0.30,0.50)	<0.001	0.93 (0.80,1.09)	0.376	0.31 (0.27,0.34)	<0.001
Sulawesi	0.40 (0.35,0.45)	<0.001	0.51 (0.39,0.66)	<0.001	0.62 (0.55,0.71)	<0.001	0.34 (0.31,0.38)	<0.001
Maluku and Papua	0.17 (0.14,0.20)	<0.001	0.10 (0.08,0.12)	<0.001	0.26 (0.22,0.31)	<0.001	0.16 (0.14,0.19)	<0.001
Urbanization								
Urban	1.00		1.00		1.00		1.00	
Rural	0.91 (0.83,1.00)	0.055	0.72 (0.60,0.86)	<0.001	0.58 (0.53,0.64)	<0.001	0.42 (0.39,0.45)	<0.001
Enabling								
Wealth status								
Poor	1.00		1.00		1.00		1.00	
Middle	1.90 (1.70,2.13)	<0.001	2.50 (1.98,3.15)	<0.001	2.51 (2.24,2.81)	<0.001	1.61 (1.49,1.74)	<0.001
Rich	2.90 (2.56,3.27)	<0.001	4.60 (3.45,6.13)	<0.001	4.23 (3.71,4.83)	<0.001	2.53 (2.33,2.74)	<0.001
Health insurance coverage								
Noncovered	1.00		1.00		1.00		1.00	
Covered	1.41 (1.29,1.53)	<0.001	1.73 (1.48,2.02)	<0.001	1.27 (1.17,1.38)	<0.001	1.28 (1.20,1.36)	<0.001
Need								
Ever had a terminated pregnancy								
No	1.00		1.00		1.00		1.00	
Yes	1.13 (1.01,1.28)	0.034	1.16 (0.94,1.42)	0.168	0.96 (0.85,1.07)	0.439	1.08 (0.99,1.18)	0.087
Number of parities								
> 2	1.00		1.00		1.00		1.00	
≤ 2	0.96 (0.89,1.05)	0.393	1.11 (0.96,1.28)	0.164	1.01 (0.93,1.09)	0.824	1.03 (0.97,1.10)	0.283

AOR, adjusted odds ratio; CI, confidence interval; JKN, Jaminan Kesehatan Nasional.

In relation to predisposing factors, as Table 3 shows, compared with reference groups, women aged 25–29, and 30–34 had a higher likelihood of utilizing ANC4, while those aged 35–39 were more likely to utilize SBA and FBD. Additionally, women with higher education levels were more inclined to utilize all four MHS services in both phases. Living in regions other than Java was associated with a reduced likelihood of utilizing all four MHS services. Moreover, residing in a rural area was linked to a reduced likelihood of accessing all four MHS services. Regarding enabling indicators, women with middle and rich wealth status and with health insurance coverage were more likely to use all four MHS services. As for need indicators, women with a history of terminated pregnancies were more likely to utilize SBA.

**Figure 2:**
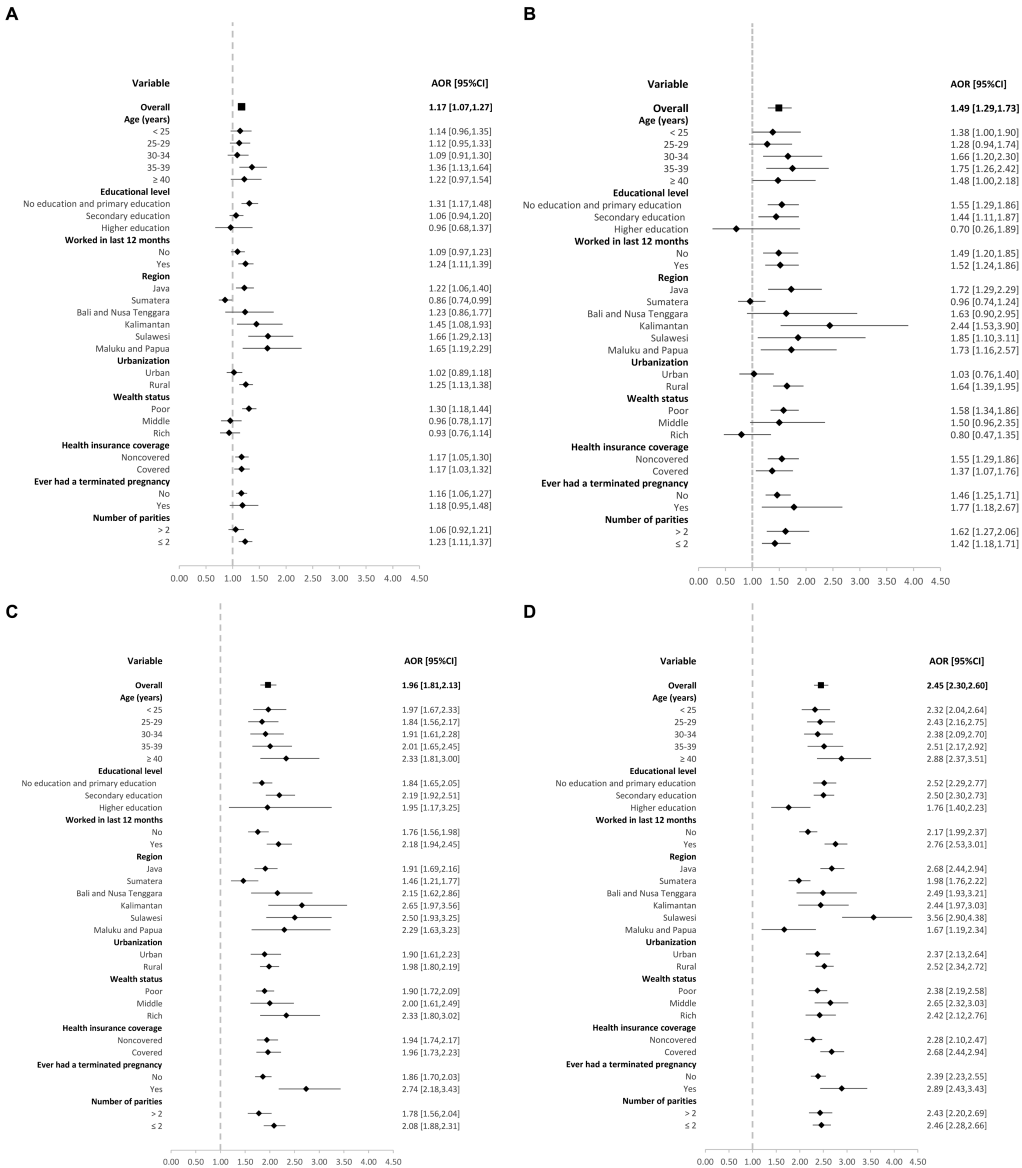
Forest plot of subgroup stratification analysis of the effect of the JKN program implementation on four maternal health services utilizationby each predisposing, enabling and need variables: **(A)** at least four antenatal care visits **(B)** skilled antenatal care provider **(C)** skilled birth assistance **(D)** facility-based delivery.

Figure 2 further provides a summary of the stratification results for the overall and policy factor effects on the four types of maternal health services examined in this study. These results are based on subgroups related to predisposing, enabling, and need factors. The findings indicate a significant overall increase in the likelihood of Indonesian women accessing all four MHS services after the implementation of the JKN. However, this effect may not be significant for women with higher education levels, those residing in the Sumatera region, and those living in urban areas. Additionally, the effect may not be significant for ANC4 among women with rich and middle wealth status, as well as for SANCP among women with rich wealth status.

## Discussion

This study compared MHS utilization before (Phase 1 in 2012) and after (Phase 2 in 2017) the implementation of JKN. The findings revealed a significant increase in Indonesian women’s likelihood to access all four MHS components: having at least four antenatal care visits, receiving care from skilled antenatal care providers, obtaining skilled birth assistance, and accessing facility-based delivery. These findings align with another research conducted in Indonesia, which also highlighted improvements in MHS utilization (26). Following the implementation of the JKN program, our results are also consistent with a study conducted across low and low middle income countries, which suggested that universal health insurance programs can have beneficial effects on MHS utilization (25, 27). The prenatal care contributes significantly to reducing maternal and neonatal morbidity and mortality, making it an essential component of comprehensive MHS. Furthermore, an increase in the number of antenatal care components received by women corresponds to a higher likelihood of them delivering at a health facility and attending postnatal care services (28).

Our study used the Anderson behavioral model as conceptual framework to access MHS utilization revealed noteworthy findings. The analysis of predisposing, enabling, and need indicators in subgroups revealed a consistent positive correlation across all indicators with a higher likelihood of utilizing MHS. In terms of predisposing indicators, specific age groups were associated with increased utilization of prenatal care. For instance, women aged 35–39 years had the highest likelihood of receiving antenatal care visits, care from skilled antenatal care providers, while women aged over 40 years had the highest likelihood of receiving skilled birth assistance and accessing facility-based delivery. This finding is consistent with earlier studies conducted in the USA and Mexico (29, 30).

The involvement of well-educated women significantly influences the choice to utilize MHS, but less impact from the implementation of the JKN program. Consistent with Laksono et al. (31) study, our results indicated the JKN program may have a prominent decrease in magnitude of socioeconomic inequality with respect to education factors. These women tend to engage in higher-paying jobs, affording them the ability to manage medical costs. Furthermore, their education empowers them with an improved understanding of their fundamental human rights and boosts their health literacy (32). As a result, it becomes crucial to improve the education system and community engagement in order to elevate the utilization of MHS (33). This study found that lower education levels had a negative impact on increasing the likelihood of utilizing multiple MHS components through the JKN program. Lower educational attainment is often associated with lower socioeconomic status, which can lead to financial constraints. With the government’s initiatives to enhance maternal and child health and make healthcare more affordable, women with lower education levels may be more inclined to engage in prenatal care. This, in turn, results in a greater reliance on public health services and an increased dependence on healthcare advice from professionals (34).

Regarding to the region factors, the government’s implementation of the village midwife program, combined with the inclusion of communities already covered by regional health insurance (Jamkesda), led to a significant increase in their utilization of healthcare services (35). Despite the overall improvement in MHS utilization across different regions of Indonesia due to the implementation of JKN, the national health insurance scheme has not reached its full potential compared with Java region (36). Notably, our stratification analysis findings indicated that women residing in Sumatera experienced a lower impact on their likelihood of utilizing MHS as the results of the implementation of JKN program. A study conducted in the Sumatera region identified predisposing factors contributing to challenges in MHS utilization. These factors included the respondent’s knowledge and attitude toward MHS, the influence of family members in healthcare decisions, and community beliefs related to MHS that did not align with health-related values (37).

In terms of enabling indicators, household wealth status, and health insurance coverage status demonstrated a tendency toward a higher likelihood of utilizing MHS. The core objective of UHC predominantly centers around addressing inequalities in healthcare among the population, particularly focusing on reaching the most vulnerable group. The current study consistent with findings from Bangladesh, which indicated that women from wealthier households exhibited a propensity to utilize antenatal care (38). Poor wealth status might be linked with a greater need for healthcare services due to a higher prevalence of health risks and conditions associated with poverty. Nevertheless, our study findings affirm that women with lower household wealth index exhibited an increase in the utilization of all four MHS services after the implementation of the JKN. Furthermore, the current study found an increasing number of any types of health insurance coverage from 36.29% in 2012 to 58.64% in 2017 after the implementation of the JKN among study subjects. It may increase accessibility to financial incentives through JKN programs and might contribute to an elevated likelihood of utilizing antenatal care (31).

As to the need indicators, women who had never experienced pregnancy termination and had a parity status of less than 2 times displayed a significantly higher likelihood of utilizing MHS. Women who have never experienced a pregnancy termination may be more likely to utilize MHS due to a higher level of reproductive awareness. They may exhibit greater apprehension regarding pregnancy complications and, as a result, seek the assistance of trained healthcare professionals more diligently (39). Additionally, women with lower parity may have more time and resources at their disposal to prioritize their own health and the health of their child. In line with this, as the fourth most populous country globally, Indonesia initiated a family planning program focused on fertility reduction through a two-child policy in 1957. This program was institutionalized in 1970 with the establishment of the National for Coordination of Family Planning (*Badan Koordinasi Kelarga Berencana Nasional, BKKBN*) (40). In some countries, women encountered a financial strain when accessing MHS and are additionally required to cover certain unofficial charges, even in the presence of formal exemptions (41). This issue becomes particularly significant if they have a larger number of children, as it contributes to an escalation in the overall incurred costs.

The primary strength of this study is its extensive national dataset, which enhances its potential for generalizability and informs policymaking at the country level. However, there are limitations that should be acknowledged. First, despite the focus on the most recent births, there is a potential for recall bias, as women were required to recall events within the preceding 5 years before the survey. Second, the associations observed might be underestimated, as only surviving mothers were available for interviews, potentially excluding uninsured women who lacked antenatal and delivery care and experienced fatal childbirth complications. Third, reliance on self-reported data introduces the possibility of under- or over-reporting certain issues. Fourth, as this analysis is based on secondary data, some well-recognized predictors of service utilization, such as cultural beliefs and family factors, are absent from our evaluations. Fifth, the cross-sectional nature of the study design used makes it impossible to establish causality. Finally, the study did not collect data on healthcare usage and out-of-pocket payments, limiting the investigation of the effects of health insurance ownership.

## Conclusion

This study compared maternal health service (MHS) utilization before (Phase 1 in 2012) and after (Phase 2 in 2017) the implementation of JKN, revealing a significant positive impact of JKN on enhancing MHS utilization. The introduction of universal health insurance coverage likely reduced financial barriers for specific demographics, resulting in increased service utilization. The study’s stratification analysis, which includes various characteristic subgroups, provided a deeper understanding of maternal health services utilization. Our study may offer valuable insights for Asian countries with similar demographics and health insurance implementations.

## Data availability statement

Publicly available datasets were analyzed in this study. This data can be found here: the DHS survey datasets, accessible through an online application request for research and study purposes, have been approved by the ICF International Institutional Review Board. Each request for data access is considered for a specific research project (http://www.measuredhs.com).

## Author contributions

TR: Conceptualization, Data curation, Formal analysis, Investigation, Methodology, Resources, Software, Visualization, Writing – original draft, Writing – review & editing. H-MH: Conceptualization, Investigation, Methodology, Project administration, Supervision, Validation, Visualization, Writing – original draft, Writing – review & editing.

## Footnotes

^1^ http://www.measuredhs.com
